# Targeting strategies for improving the efficacy of nanomedicine in oncology

**DOI:** 10.3762/bjnano.10.16

**Published:** 2019-01-14

**Authors:** Gonzalo Villaverde, Alejandro Baeza

**Affiliations:** 1 Dpto. Química Inorgánica y Bioinorgánica, Universidad Complutense de Madrid, Instituto de Investigación Sanitaria, Hospital 12 de Octubre i+12, Plaza Ramón y Cajal s/n, 28040 Madrid, Spain; CIBER de Bioingeniería, Biomateriales y Nanomedicina, CIBER-BBN, Madrid, Spain; 2 Dpto. Materiales y Producción Aeroespacial, ETSI Aeronáutica y del Espacio, Universidad Politécnica de Madrid, 28040-Madrid, Spain

**Keywords:** antitumoral therapy, nanomedicine, smart nanocarriers, targeted nanoparticles

## Abstract

The use of nanoparticles as drug carriers has provided a powerful weapon in the fight against cancer. These nanocarriers are able to transport drugs that exhibit very different nature such as lipophilic or hydrophilic drugs and big macromolecules as proteins or RNA. Moreover, the external surface of these carriers can be decorated with different moieties with high affinity for specific membrane receptors of the tumoral cells to direct their action specifically to the malignant cells. The selectivity improvement yielded by these nanocarriers provided a significative enhancement in the efficacy of the transported drug, while the apparition of side effects in the host was reduced. Additionally, it is possible to incorporate targeting moieties selective for organelles of the cell, which improves even more the effect of the transported agents. In the last years, more sophisticated strategies such as the use of switchable, hierarchical or double targeting strategies have been proposed for overcoming some of the limitations of conventional targeting strategies. In this review, recent advances in the development of targeted nanoparticles will be described with the aim to present the current state of the art of this technology and its huge potential in the oncological field.

## Introduction

Nanotechnology has become a powerful weapon in the search of novel strategies for addressing unmet clinical challenges, from the treatment of complex diseases as cancer or neurological disorders, to the early diagnosis of these pathologies that could allow for eliminating them before the appearance of any symptoms. Nanoparticles can interact with cells, bacteria and viruses in a very intimate and efficient way because they present a similar size than these biological entities [1]. This close interaction has been exploited for achieving important abilities such as the selective transport of drugs directly to diseased cells and tissues [2], the precise recognition of extremely low concentrations of important biomarkers indicative of pathological processes that are present in complex environments (e.g., urine, blood, saliva) [3] or the creation of smart nanorobots able to perform precision surgery inside the body [4]. One of the applications in which the nanoparticles have found an interesting niche is oncology. The conventional treatment of cancer is based on three strategies: surgery, radiotherapy and chemotherapy. These approaches exhibit a lack of selectivity affecting also the surrounding healthy tissues, in the case of surgery and radiotherapy, and/or to the whole organism, in the case of chemotherapy. This last strategy could be visualized as carpet bombing with the aim of destroying an enemy army that is hidden in a populated city. In many cases, the effect on the malignant “soldiers” is scarce but the number of “civilian casualties” is unbearable. Nanomedicine has provided a promising alternative to these strategies through the development of engineered nanocarriers capable to deliver therapeutic agents specifically to tumoral cells without affecting healthy tissue. These nanoparticles are able to load great amounts of drugs, to transport them in the blood stream and finally, to recognize the tumoral tissue and release their cargo inside the tumoral cells. The idea to use nanoparticles as drug carriers in oncology arose in 1986, when two Japanese researchers reported that nanoparticles present a passive tendency to be accumulated into tumoral tissues [5]. This passive accumulation, also known as passive or primary targeting, is called “enhanced permeation and retention (EPR)” effect and is one of the keystones of tumour treatment with the help of nanocarriers [6]. Moreover, the external surface of these nanocarriers can be decorated with different bio-organic moieties (targeting groups) that bind specifically to receptors located on the membrane of tumoral cells in order to enhance the particle uptake in the malignant cells. This strategy is the so-called “cellular or secondary targeting”, because it is generally based on a ligand–receptor-mediated endocytosis, triggered by the strong interaction of the targeting group with the membrane receptor of the tumoral cell. It improves the selectivity of the treatment achieving a drastic reduction of the side effects caused by the transported drugs and also reduces the drug resistance developed through the high doses in conventional treatments [7]. Finally, it is also possible to place additional targeting moieties on the particle surface that do not bind to receptors located on the external membrane of the cells but recognize internal organelles. This approach is known as tertiary targeting and it has been widely exploited for the transportation of potent cytotoxic compounds or genetic materials (i.e., silencing RNA) that present an improved effect when they are released close to specific organelles such as mitochondria or the nucleus. In this review, some of the recent advances of the different targeting approaches investigated in the last years will be presented. Additionally, the development of sophisticated strategies that allow for the sequential targeting of cells and organelles, or tissues and cells, as well as the employ of hierarchical targeting will also be described to provide an insight about the great potency of targeted nanomedicines in antitumoral therapy.

## Review

### Passive targeting based on the EPR effect

**Figure 1:**
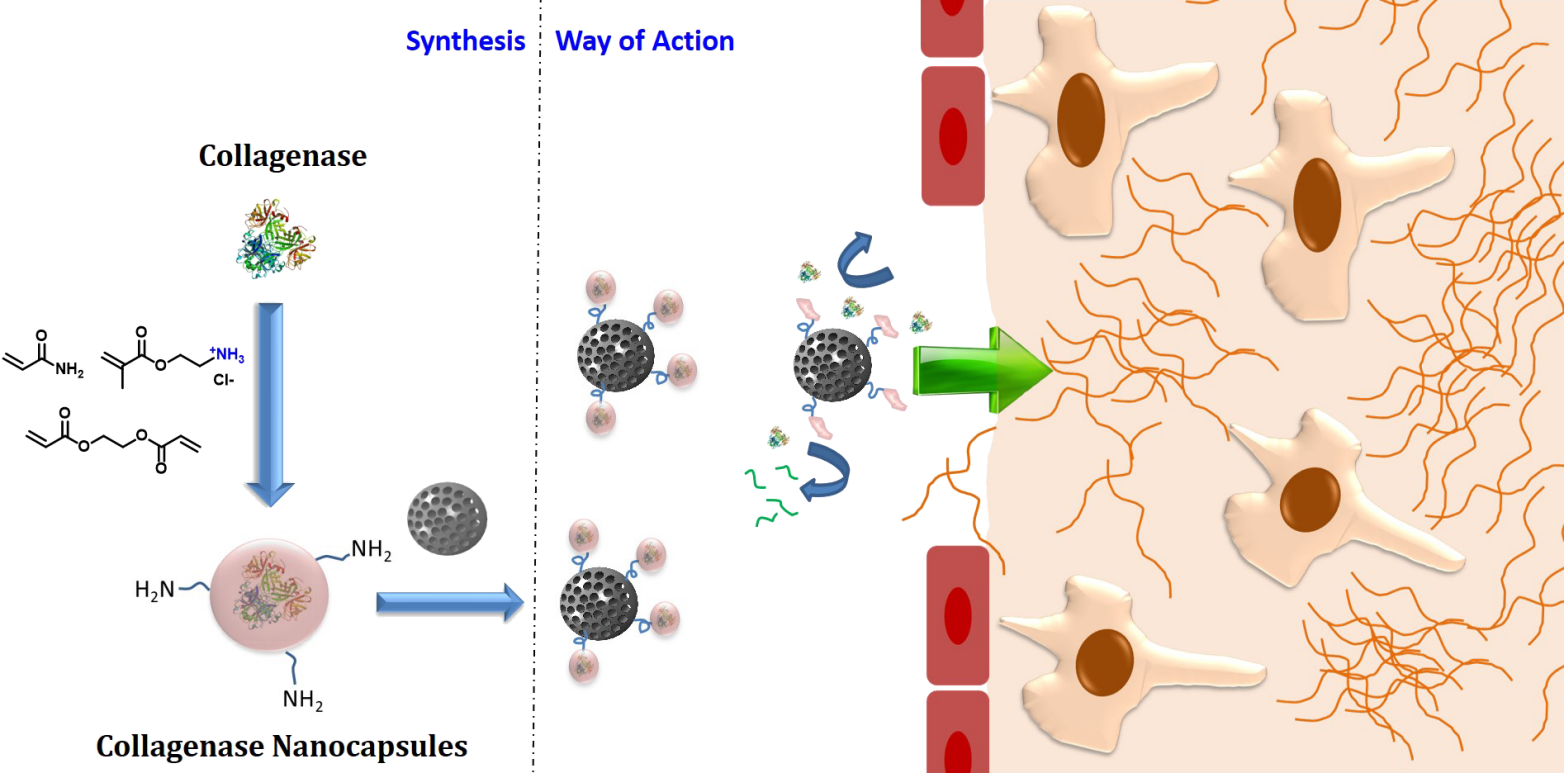
Highly penetrating nanosystems based on the incorporation of pH-responsive collagenase nanocapsules. This image has been adapted from [15], copyright 2015 American Chemical Society.

As mentioned above, the use of nanoparticles in oncology was proposed for the first time by Maeda and Matsumura, who reported the selective accumulation of nanometric entities in tumoral tissue [5]. The reason of this passive accumulation lies in the unique architecture of the blood vessels that irrigate the solid tumour. The accelerated growth of a solid tumour must be sustained by the continuous construction of blood vessels in order to transport nutrients and oxygen to the malignant cells spreading through the tissue. The creation of completely functional blood vessels requires a fine balance between pro- and anti-angiogenic factors. These factors are unbalanced in the tumoral tissue with the amount of pro-angiogenic factors being higher [8]. As a consequence of this, the newly formed blood vessels have an aberrant and tortuous structure with pores and fenestrations of a few hundreds of nanometres. Therefore, when the nanoparticles reach the tumoral blood vessels, they can leak from the vessels through these pores into the malignant tissue. Moreover, the accelerated growth of the tumoral mass usually compresses the lymphatic vessels that are on charge of the elimination of wastes products and liquids from the tissue and thus, the extravasated nanoparticles cannot leave the tissue resulting in accumulation over long periods of time. These two characteristics, the high permeability of tumoral blood vessels and the lack of an efficient drainage system are responsible for the accumulation of the nanoparticles into neoplastic tissues. Unfortunately, the EPR effect is not as universal as originally thought. It highly depends of the type of tumour and even of the state of disease progression [9]. Despite the fact that the EPR effect is really pronounced in mice models, this effect is not general in humans. There are tumours with a very pronounced EPR effect, such as Kaposi sarcoma and multiple myeloma, while other tumours barely exhibit this effect, as pancreatic cancer. Therefore, it is required to design strategies able to increase the nanoparticle accumulation in tumoral tissues where the EPR effect is weak [10]. Additionally, even in the case where the EPR effect is present, there are other barriers that compromise the efficacy of nanoparticle-based therapies. One of these barriers is the elevated interstitial fluid pressure (IFP) present in the interstitial space of solid tumours, which approaches or even surmounts the intravascular pressure [11]. This effect strongly compromises the diffusion of the nanoparticles into the tumour tissues. Some authors have proposed the previous normalization of the tumoral vasculature by the administration of anti-angiogenic factors in order to reduce the IFP and therefore, to enhance the nanoparticle diffusion into the tumour [12]. Another strong barrier that hampers the efficacy of nanomedicines is the dense extracellular matrix (ECM), which is usually present in many solid tumours. ECM is commonly denser in solid tumours than in healthy tissues due to a higher content in collagen and other structural proteins. This fact hinders the penetration of the nanoparticles into tumoral tissues restraining their effect to the periphery of the neoplasia. In order to overcome this limitation, diverse alternatives have been proposed, from the application of ultrasounds for propelling the nanoparticles inside the tissue [13] to the previous administration of proteolytic enzymes that digest the ECM [14]. As an example, Villegas et al. have reported the use of pH-sensitive polymeric nanocapsules that are able to release collagenase once they arrive at the tumoral tissue due to the mild acidic conditions present there [15]. These nanocapsules were anchored on the surface of mesoporous silica nanoparticles (MSN) coated with a lipid bilayer (protocells) enhancing their penetration into 3D tumoral tissue models, which yielded a significant enhancement of the therapeutic efficacy of these nanodevices (Figure 1) [16].

### Active targeting: from cellular to organelle vectorization

**Figure 2:**
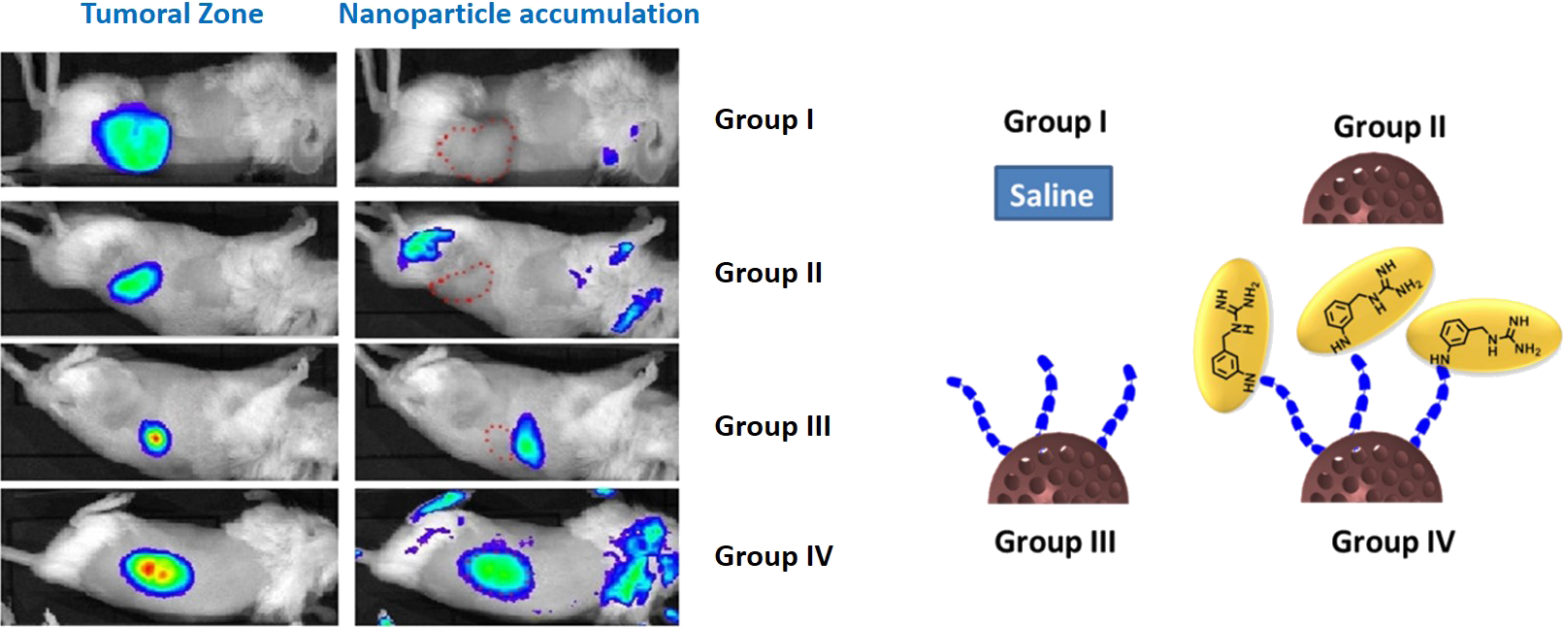
Left: tumoral zone monitored by bioluminescence and nanoparticle accumulation detected by IVIS^®^ Spectrum in vivo imaging system. Group IV exhibited strong particle accumulation in the tumour, whereas group III only showed slight accumulation in the liver. Right: images of each group of particles that were injected in mice. This image has been adapted from [23], copyright 2015 Royal Society of Chemistry.

Once the nanoparticle reaches the tumoral area, it faces a complex scenario. Tumoral masses are not composed by an homogeneous tumoral cell distribution but they are formed by a myriad of different cell populations, from tumoral cells to immune, supportive and healthy cells of the original tissue [17]. Therefore, nanoparticles should possess the capacity to recognize the malignant cells and focus the effect onto them in order to achieve an efficient therapeutic effect. This ability can be incorporated in the nanodevice by anchoring targeting moieties on the particle surface [1,18]. These targeting moieties are molecules or macromolecules that bind to specific receptors located on the surface of the tumoral cells. In many cases, these cellular receptors are also present in healthy cells, e.g., for the widely employed targeting moieties folic acid [19], transferrin [20] or sugars [21]. But their number is significantly higher in tumoral than in healthy cells due to their stronger nutrient demand. Thus, this receptor overexpression can be exploited for the selective delivery of therapeutic drugs to tumoral cells. Another possibility consists in the development of synthetic targeting moieties that bind to certain receptors in a more selective and efficient manner [22]. Villaverde et al. have reported the synthesis of *meta*-aminobenzylguanidine (MABG) and its anchorage to the surface of MSN in order to guide these particles specifically to neuroblastoma cells [23]. About 90% of neuroblastoma cells overexpress the norepinephrine receptor (NET) on their surface. *Meta*-iodobenzylguanidine is a synthetic analogue of norepinephrine that, with a radioactive iodine substituent (^131^I), has been widely employed for the diagnosis of neuroblastoma due to its strong affinity for NET. The replacement of the iodine by an amino group in MABG did not reduce the ability to bind to NET while it provided a reactive group that allowed for grafting this molecule to the surface of MSN employing a bifunctionalized polyethylene glycol (PEG) molecule as spacer between the MSN surface and MABG. MSN decorated with these moieties were engulfed by neuroblastoma cells up to four times more than non-targeted MSN. The in vivo evaluation in neuroblastoma xenograft model showed strong accumulation of the targeted system and high retention in the tumoral zone over a period of more than 72 h. Non-targeted nanoparticles were rapidly cleared. Interestingly, MSN decorated with the same type of PEG but without MABG at the end failed to be accumulated in the tumoral area, but they showed slight accumulation in the liver of the animal, probably due to the increase in the circulation time of the particles caused by the presence of the PEG chains (Figure 2) [23].

The small size of these synthetic molecules allows for the grafting of multiple copies of them, or even combinations of two different molecules on the surface of the nanoparticles [24]. This fact can induce a significant enhancement of the particle uptake due to multiple binding processes with tumoral receptors through a multivalence effect [25]. Nature usually employs antibodies for the recognition of cells and pathogenic bodies. Antibodies are large proteins that present a characteristic Y-shaped structure in which the recognition event takes place in a very specific manner through the interaction between the antigen located on the diseased cell and the two ends of the Y-shaped protein. Thus, many different antibodies have been attached on the surface of multiple types of nanoparticles to induce selectivity against specific cell populations. As example, Herceptin is an antibody that recognizes the human epidermal growth factor receptor 2 (HER2) overexpressed in breast cancer cells (HER2+). This antibody has been attached on the surface of poly(D,L-lactic-*co*-glycolic acid) (PLGA) nanoparticles loaded with the potent estrogen receptor modulator tamoxifen [26]. These nanoparticles were capable to induce a significant in vivo tumour growth inhibition due to the enhanced nanoparticle uptake within the tumoral cells owing to the strong interaction between the antibody and HER2. Another interesting possibility is to employ antibodies for the recognition of the tumoral blood vessels instead of the tumoral cells. Endoglin, or CD105, is a glycoprotein usually overexpressed on the surface of the endothelial cells which compose the tumoral vessels. TRC105 is a human/murine chimeric antibody which recognizes CD105 with high specificity and due to this property it has been incorporated on the surface of MSN labelled with ^64^Cu in order to perform imaging by positron emission tomography (PET) [27]. The injection of these particles into the blood stream of mice bearing breast tumour allowed for the visualization of the tumoral mass thanks to the enhanced accumulation of the particles. One of the problems associated with the use of antibodies for targeting is the partial loss of the binding capacity of the attached antibody during the anchoring process, which is usually carried out by non-specific chemical techniques as carbodiimide coupling chemistry [28]. An alternative is the introduction of certain functional groups on specific positions of the antibody, which allows the utilization of bio-orthogonal chemical strategies, such as azide-strained alkyne or thiol–maleimide reaction, for carrying out the antibody attachment with a high precision level [29]. Another interesting strategy is the attachment of protein A on the nanoparticle surface prior to the incorporation of the antibody [30]. Protein A is a membrane protein produced by *Staphylococcus aureus* in order to complex the immunoglobulins by the complement region (Fc) deactivating the immune attack of the host. Thus, antibodies can be anchored to the surface of a nanoparticle decorated with protein A thanks to the strong affinity between the protein and the Fc region, which is not involved in the recognition process and therefore, this process occurs without any loss of the antibody binding capacity. Unfortunately, the use of antibodies as targeting moieties has an important drawback, which is the possibility to trigger immune responses due to the uncontrolled exposition of immunogenic regions (as Fc) on the particle surface. Peptides are versatile alternatives to antibodies for targeting purposes. The use of relatively short peptide chains provides some important advantages, such as i) only little alteration of the hydrodynamic diameter of the nanoparticles, ii) multigram production with high purity, iii) possibility to attach multiple copies of them on the nanoparticle surface which enhances the uptake, iv) possibility to use non-natural aminoacids improving the versatility and v) low immunogenicity [31]. The tripeptide Arg–Gly–Asp (RGD) is probably one of the most employed peptide in the targeting design of nanoparticles. RGD binds specifically to αβ-integrin, which is usually upregulated in many different tumoral cell lines such as breast, lung or fibroblast cancer cells, and also by the epithelial cells of the tumoral blood vessels [32–33]. Ruoshlati et al. have reported that the cyclic version of RGD, CRGDKGPDC (called iRGD), which is cyclized by the disulfide bridge between both terminal cysteines, exhibits significantly a higher tumour specificity than linear RGD [34]. iRGD works in a sequential manner, first it binds to αβ-integrin by the RGD sequence encrypted within the cyclic structure and then, the peptide is broken by the action of a cell surface-associated protease exposing the RGD, which then binds to neuropilin-1 triggering the particle endocytosis. Another cell-penetrating peptide (or CPP, which is the usual name of the peptides used for targeting purposes in nanomedicine) closely related to RGD is the tripeptide Asn–Gly–Arg (NGR). The asparagine present in this peptide sequence experiences spontaneous deamidation producing a mimetic of the RGD peptide (iso-DGR), which presents similar targeting capacities. Additionally, this sequence also binds to tumoral blood vessels [35]. Thus, this peptide has been anchored to different nanoparticles for enhancing their uptake into tumoral cells or for binding to tumour vessels. As an example, cyclic NGR, which binds to the aminopeptidase receptor (CD13), was grafted on the surface of temperature-sensitive liposomes loaded with doxorubicin (Dox) for the selective destruction of CD13+ cancer cells as human fibrosarcoma cells (HT-1080) [36]. These liposomes released more than 75% of their payload when the temperature reached 41.3 °C whereas they maintained the Dox within their hydrophilic core at physiological temperature. Other systems widely employed for targeting purposes are aptamers. Aptamers are oligonucleotide chains that exhibit a characteristic three-dimensional structure capable to bind to specific membrane cell receptors overproduced by the tumoral cells. The aptamer that specifically binds to a certain protein is usually selected by the technique named systematic evolution of ligands by exponential enrichment (SELEX) [37]. Through this technique it is possible to obtain oligonucleotide sequences selective for many different membrane proteins. These macromolecules have been widely employed both alone and conjugated with drugs or nanoparticles [38]. Aptamers specifically designed for binding to the epidermal growth factor receptors (EGFR) have been anchored on the surface of hollow gold nanospheres [39]. The thiolated version of these aptamers was anchored on the gold surface through the thiol groups producing an average anchorage yield of 250 aptamers per particle. The biodistribution of these particles was evaluated in vivo by micro-single-photon emission computed tomography/computed tomography (micro-SPECT/CT) employing particles labelled with ^111^In, showing an excellent tumour-homing capacity of these particles. AS1411 aptamers have been widely employed for cell targeting in tumoral cell lines that overexpress nucleolin [40].

The use of targeting moieties provides not only the capacity to the nanoparticles to be selectively engulfed by tumoral cells. It also allows for the localization of the nanocarriers in specific intracellular localizations or organelles, such as nucleus or mitochondria [41–42]. This enables the precise delivery of therapeutics to key organelles of the cells, which could significantly increase their cytotoxic effect. Mitochondria are the energetic plants of the cells. In addition, they carry out other important functions such as the control of the intracellular calcium concentration or the removing of the oxidative species, which could damage the cell. Therefore, the specific delivery of toxic species to these key organelles compromises the function of the entire cell causing its destruction. Yoong et al. have decorated the external surface of multiwalled carbon nanotubes (MWCNTs) with rhodamine-110 to localize them close to the mitochondria membrane [43]. The positive charge provided by rhodamine-110 provokes the electrostatic binding with the highly negative mitochondria membrane (−180 mV to −160 mV). These MWCNTs were loaded with a platinum(IV) pro-drug that released active cisplatin(II) in the reductive environment of the intracellular space. Another targeting moiety that has been employed for delivering therapeutics to mitochondria is triphenylphosphine [44]. This positively charged group also binds to the mitochondria membrane by electrostatic interactions. The nucleus contains practically all the genetic information (except for the mitochondrial DNA) and is of paramount importance for the correct function of the entire cell. Targeting nuclei has received huge attention regarding the delivery of cytotoxic species that act on DNA or the direct delivery of genes to their place of action. Viruses are one of the inspiration sources for strategies to reach the inner nuclear space. They contain on their membrane small peptide sequences with nuclear translocation capacity such as the KKKRKV peptide in simian vacuolating virus 40 (SV40), GRKKRRQRRRPQ in the TAT peptide present in human immunodeficiency virus (HIV), or KRPAATKKAGQAKKKKL in the case of nucleoplasmin [45]. These peptides have been anchored on the surface of different nanocarriers providing excellent results [46]. The aptamer AS1411 selective for nucleolin, a protein present on the nuclear membrane, has also been widely employed for the selective release of therapeutic compounds to the nucleus [47–48]. Shiga and cholera toxins exhibit the ability to target Golgi and endoplasmic compartments and they have been conjugated with drugs for their selective delivery to these organelles [49]. The peptide Lys–Asp–Glu–Leu (KDEL) has been anchored on gold nanoparticles loaded with siRNA for the selective delivery of the genetic material into the endoplasmic reticulum [50].

The main mechanism for the internalization of nanoparticles within mammalian cells is endocytosis [51]. Usually, the nanocarriers enter into the cells into endosomes, which evolve into lysosomes, which can lead to the degradation of the transported cargo, especially in the case of sensitive agents such as genes or siRNA. Therefore, it is necessary to design mechanisms to induce the endosomal escape to reach the cytosol. Multiple strategies for overcoming the endosomal entrapment have been designed [52]. One of the most widely employed is the incorporation of polycationic groups on the particle surface such as poly(ethyleneimine), cationic dendrimers or poly(histidine) chains [53]. Tertiary amino groups in these polymers bring protons into the endosomes producing osmotic alterations that provoke endosomal rupture (proton sponge effect) [54]. The incorporation of peptides such as the GALA peptide (WEAALAEALAEALAEHLAEALAEALEALAA) capable to fuse with the endosomal/liposomal membrane is another mechanism for inducing endosomal escape of nanomedicines [55]. Finally, the incorporation of photosensitizers able to produce radical oxidative species (ROS) upon exposure to certain wavelengths of light induces the controlled endosomal disruption under light exposure [56].

### Double targeting solutions, a real alternative?

Active targeting is already one of the most used strategies for bringing nanoformulations into tumoral cells. Although usually great results were achieved in vitro, the in vivo assays have shown smaller effects regarding cell internalization. There has been no real enhancement in the treatment efficacy compared to the passive vectorization effect provided by EPR [57]. Physical and also biological barriers disrupt, to a high extent, the desired selective interactions between the targeting ligands and their receptors. Effects such as off-targeting towards common cell receptors expressed in tumoral but also in healthy cells, and the rapid uptake by the reticuloendothelial system, macrophages and supportive cells such as fibroblasts the decrease the nanocarrier concentration in the blood stream. Also, the poor penetration capacity into the tumoral mass due to strong interactions between cell receptors and targeting agent in the first layers of cells of the tumoral tissues, to so-called binding-site barrier effect, reduces the efficacy of the nanomedicine to an outside stratum of the tumoral zone. In contrast, good results for imaging have been achieved to improve diagnosis in early stages of the disease. Thus, active targeting is still widely studied not only for nanomedicine but also for conjugate drugs [58–59].

As was mentioned above, there are three levels of active targeting: tissular targeting, cellular targeting and intracellular or organelle targeting. A combination of them in single system providing new functionalities and capacities may allow the system to overcome the natural barriers of the nanomedicine approaches. The improvement of the EPR effect in order to increase the nanomedicine accumulation, retention and even penetration into the diseased tissue is one of the main goals [10]. Usually, fast growing tumours such as carcinoma exhibit a highly vascularized tumour mass, while slow growing tumours as sarcoma are poorly irrigated [60]. There are three main strategies for improving the accumulation and retention in tumour tissue: i) the modification of physical conditions of the tumor mass; ii) the selectively targeting of the payload towards tumoral stroma or vasculature tissue and iii) to kill the cancer cells that belong to the external shell of the tumour primary layers [61]. All of them and their combinations need active tissular or cellular targeting systems for a better performance. The strategies for providing multiple targeting abilities within one single nanocarrier will be discussed in the following section.

### Simultaneous targeting of tissue and cells

Double vectorization has been proposed in the last years as an approach to overcome some of the physical barriers in nanomedicine. The combination of tissular and cellular targeting agents in a unique nanocarrier may improve accumulation and/or the uptake in cancer cells without affecting healthy cells.

**Figure 3:**
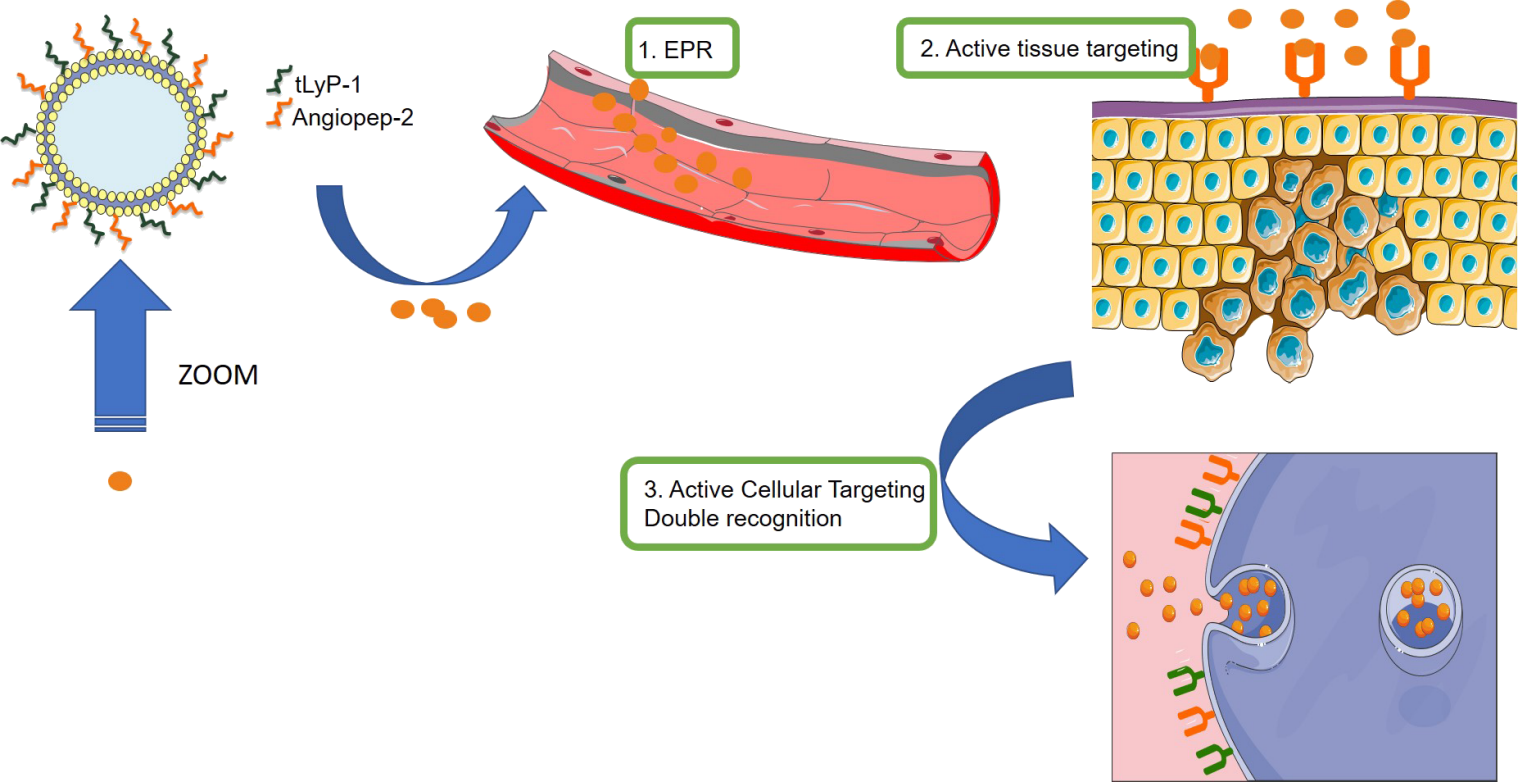
EPR and active targeting working together for glioma tissue accumulation and preferential glioma cells internalization.

Firstly, the simplest approach is to randomly attach both tissular and cellular targeting moieties on the nanocarrier surface. Through tissular targeting the nanocarrier would be directed to the diseased cells improving its accumulation. Once there, the presence of the cellular targeting moieties would enhance the cellular uptake into the tumoral cells. In several types of cancers and depending on the location of the malignant tissue, the EPR is not effective at all. Combining tissular and celluar agents is a powerful tool in such cases making that active tissular targeting ligands even more important. In 2014, Yang et al. [62] described a peptide dual targeting system with drug-loaded liposomes for glioblastoma treatment. Glioblastoma, localized in the brain, represents one of the major challenges in drug delivery due to the necessity to pass the blood brain barrier (BBB). BBB inhibits the passage of 98% of the medicines administered through the systemic route and constitutes a formidable barrier for tissue targeting not only in nanomedicine but also in common drug delivery [63]. In this case, liposomes were modified with Angiopep-2 and tLyP-1 homing and penetrating peptides for the simultaneous delivery of siRNA and (docetaxel) DTX. Angiopep-2 showed affinity to the lipoprotein receptor (LPR) typically overexpressed in glioma and in BBB cells [62] and therefore, it shows excellent capabilities for the penetration into the brain through the transcytosis pathway. The peptide tLyP-1 also exhibits both tissue penetration ability through the neurophilin-1-dependent C-end rule and affinity to glioma cells for LPR interaction. The exposed dual peptide cation enables the possible accumulation into gliomas via the combination of EPR effect and active targeting for an antiangiogenic and apoptotic treatment. In vitro assays showed improved internalization only when the liposomes have both targeting systems exposed, demonstrating the synergy of the two peptides in the assisted internalization (Figure 3). In vivo experiments showed an amazing reduction in subcutaneous induced glioma tumours in mice by intratumoral but also by systemic administration. This example represents the collaboration of two targeting agents to improve the vectorization of the system on tissular and cellular levels.

**Figure 4:**
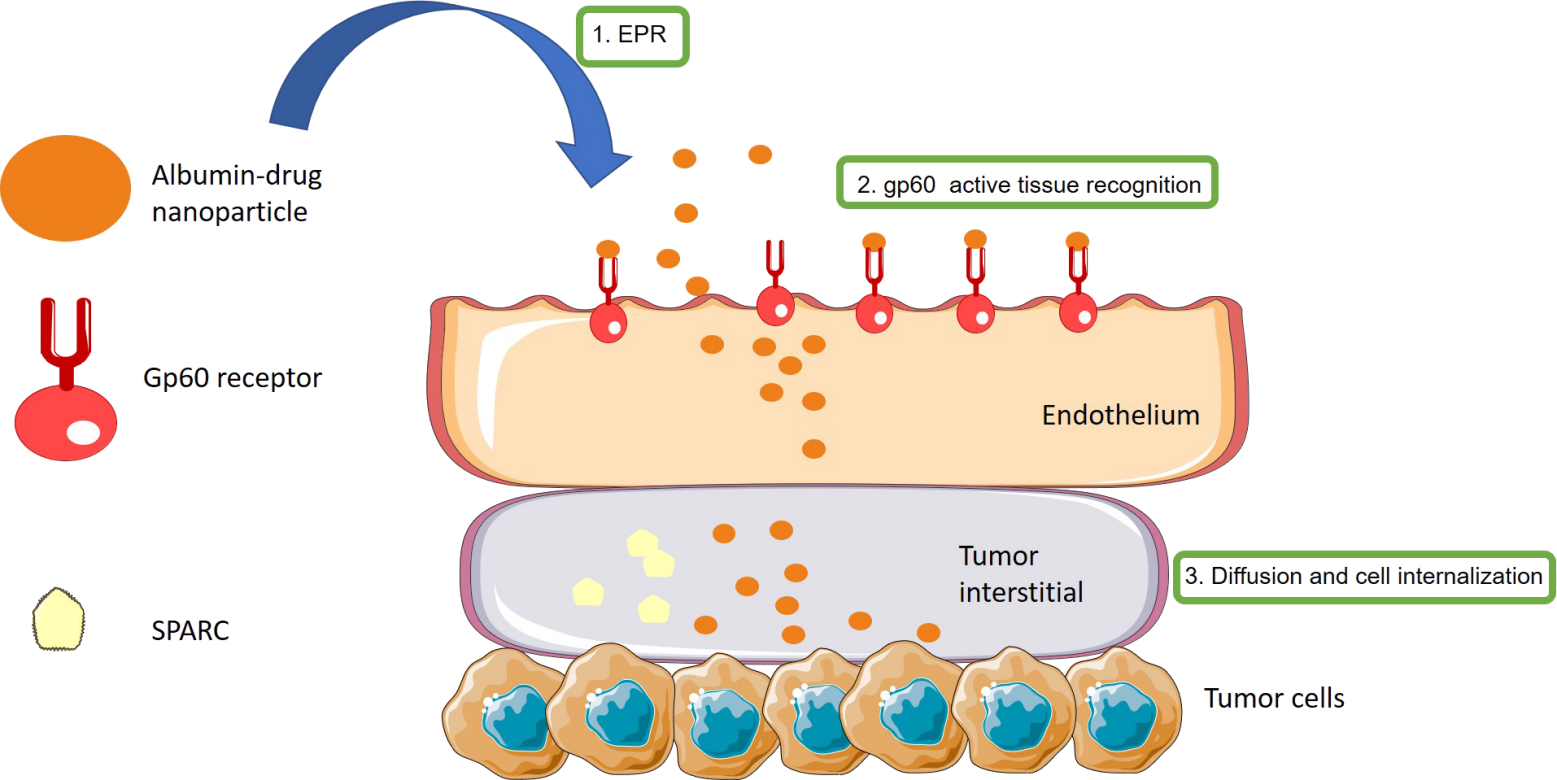
Mechanism of retention and accumulation of Abraxane in tumoral tissue. The authors’ own generalized depiction of the scheme proposed previously in [64].

Another representative example of cooperation of multiple tissular and cellular targeting systems is the use of albumin-based systems [64]. Their accumulation properties in tissue are based on two effects: the EPR due to their size and active targeting provided by the glycoprotein gp60 interaction. The protein gp60 is overexpressed in the endothelial cell surface and allows the albumin-based systems to extravasate to the tumour mass through caveola formation and transcytosis. After this, albumin may also bind to the “secreted protein, acidic and rich in cysteine” (SPARC) present in the extracellular matrix, facilitating the approximation to tumoral cells. This is the postulated mechanism of action of Abraxane^®^, one of the most commonly administered nanomedicines based on albumin today (Figure 4).

**Figure 5:**
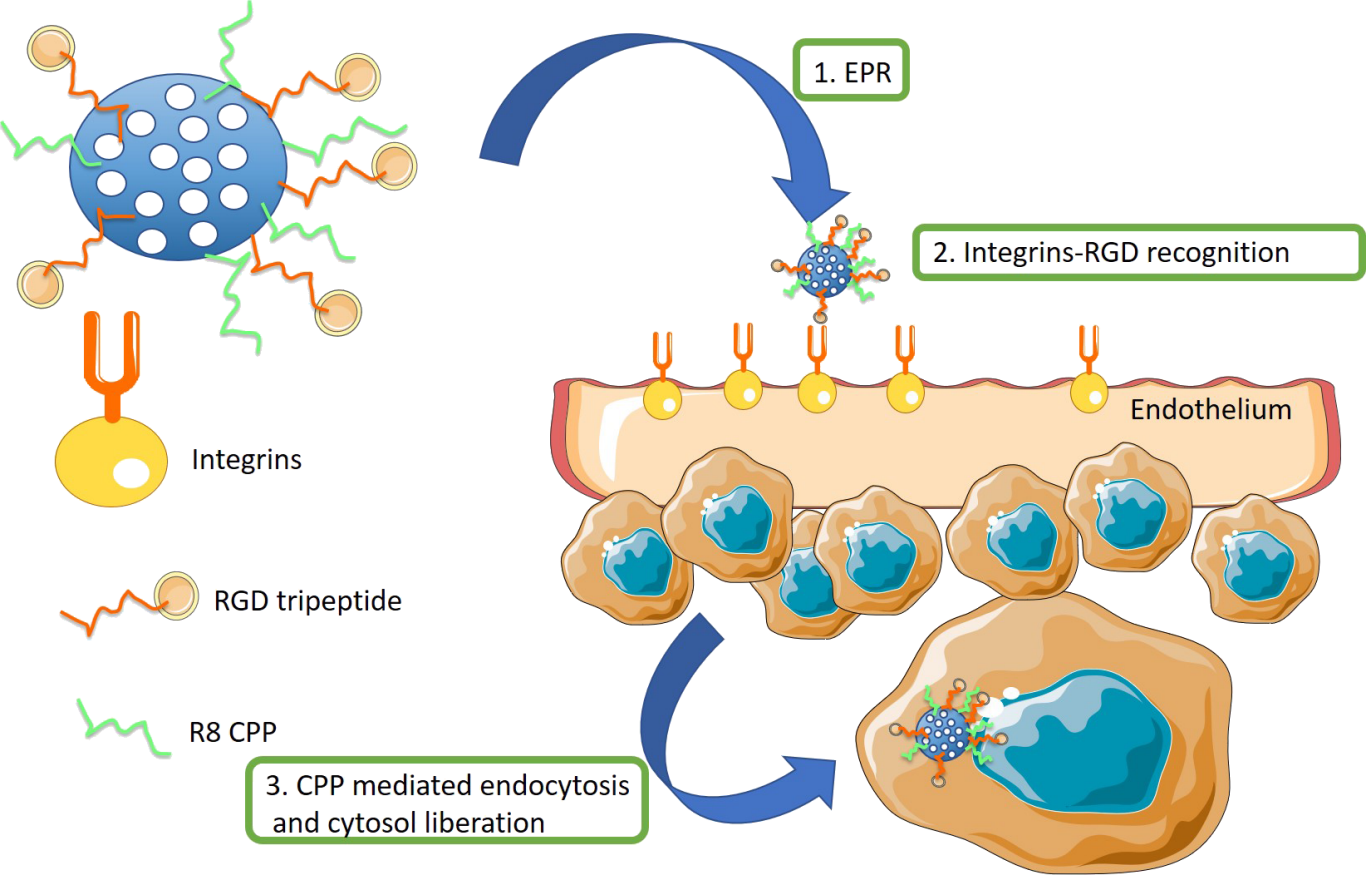
Combination of unspecific CPP and endothelial specific ligands for tissue recognition and efficient internalization on cells.

Another commonly employed strategy to improve the transportation efficiency of nanosystems is the combination of an unspecific cell penetrating peptide (CPP) with a selective targeting ligand. With this strategy, the nanocarrier combines the selectivity of the receptor–ligand interaction with the power of the CPP for an effective internalization and an endosomal escape to the cytosol (Figure 5). This methodology has been applied by using a combination of RGD-type or NGR-type peptide specific for neovascular tissues with R8 (eight units of arginine) or R4 (four units of arginine) CPP peptides [65–66].

### Hierarchical and encrypted sequential targeting – novel strategies for dual targeting

**Figure 6:**
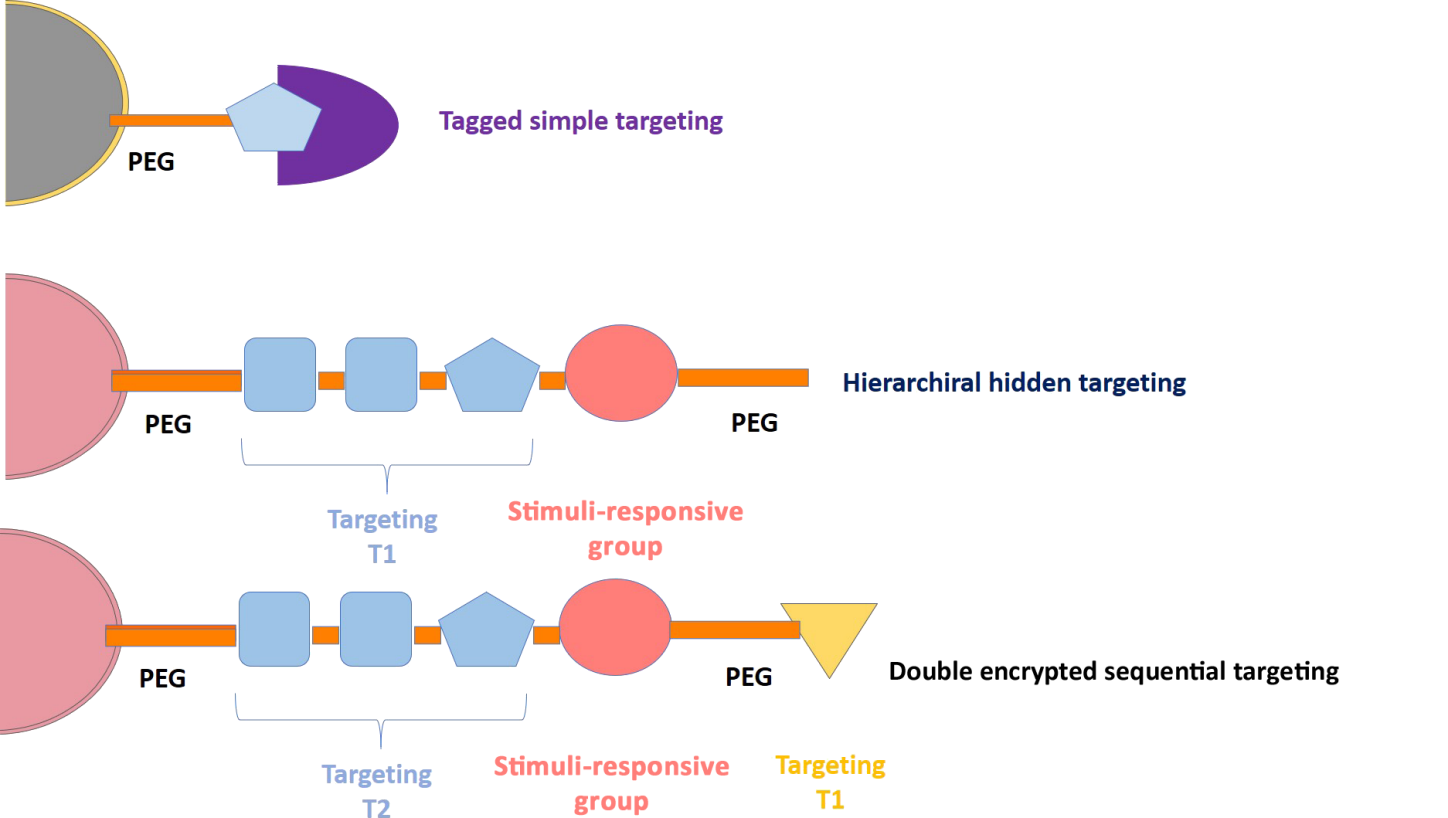
Different strategies for the construction of hierarchical targeting ligands.

All targeting methodologies described above are obviously of limited clinical use. The coexistence of two active vectorization ligands at the same time and close to each other may lead to unwanted interactions not only between them, but also with the physiological environment during circulation. Furthermore, the existence of positively or negatively charged moieties or active targeting groups on the nanocarrier surface could reduce the circulation time of the systems by off-target accumulation or accelerated clearance by the reticuloendothelial system (RES). This leads to a reduced accumulation of the nanocarriers in the diseased tissue. The concept of spatiotemporally modulated dual-targeting systems has been introduced lately as a response to these undesired interactions between two targeting motives [67]. With the aim to control and tune the targeting properties depending on time and the localization of the nanocarrier, hierarchical targeting has been recently proposed as a novel strategy [68]. This strategy is based on hiding the targeting moieties and only activating them in the appropriate scenario. Hierarchical targeting systems incorporate stimuli-responsive strategies in such a way that the targeting groups are hidden during the circulation of the carrier through the body and, therefore, the tissular accumulation occurs mainly through the EPR effect. Once the carrier reaches the tumoral tissue, the specific conditions there (e.g., low pH values or the presence of certain enzymes) or the application of an external stimuli (e.g., light, magnetic fields or ultrasound) triggers the targeting inducing the particle uptake into the diseased cells [69–71]. Thus, the targeting is only activated in the malignant tissue, which would significantly reduce the off-target accumulation and the RES clearance. The hierarchical systems can be classified according to the targeting activation mechanism and include changeable particle sizes, switchable surface charges and activatable surface ligands (Figure 6).

There are numerous active vectorization motifs that are capable of being tagged for their deactivation, from small molecules such as folic acid to penetrating (CPP) or homing peptides (RGD-type). The tagged agents may be activated once the system is accumulated in the tumoral tissue by EPR through both internal or external stimuli [72–74]. The tagging motive should be designed specifically for each type of active targeting. To solve this limiting problem of scope, one of the main strategies followed lately is the use of shielding molecules with high molecular weight such as PEG for simply sterically hindering the active vectorization agents. Usually, PEG chains are functionalized through a responsive group over the nanocarrier surface. Thus, PEG plays a dual role. It acts as shield that keeps the targeting agent hidden and it prevents the opsonisation of the nanocarrier, which would be the first step required for a capture by macrophages. When the system reaches the tumoral tissue, the PEG chain is cleaved leaving exposed the targeting ligand for tumoral cell recognition and internalization. In the recent years, several works reported that the use of PEG could induce immunogenic reactions in the host due to the production of anti-PEG antibodies. More research is required to clarify this question [75–76].

The use of shielded targeting agents renders the EPR effect [77–78] the sole reason for nanocarrier accumulation in tumoral tissues [79]. Sometimes this is not effective at all. Double sequential targeting strategies have been proposed as important alternatives. In these systems, there are two targeting agents: a primary moiety selective to the tumour tissue and a secondary moiety selective to tumoral cells but only active when the system reaches the tumoral mass. In 2017, Villaverde et al. [80] described a double sequential encrypted targeting system focused on bone tumour. Bone is usually poorly irrigated and nanoparticles have serious difficulties to reach this tissue. The system is based on a hybrid peptide/polymeric chain that contains a bisphosphonate (BP) group at the end. BP acts as tissular targeting motif, due to its high affinity for the exposed mineral part of bones (hydroxyapatite). An RGD sequence conjugated with a peptide sequence cleavable by the action of cathepsin-K (CK) was incorporated within the hybrid chain. CK is a proteolytic enzyme, which is usually typically overproduced in osteosarcoma tumours. Thus, the complete targeting moiety, which can be conjugated with drugs or nanoparticles, induced the accumulation in diseased bone tissue in which the mineral part is more exposed than in healthy bones due to the disruption of the bone architecture caused by the tumour. When the bone tissue presents a malignancy, the local overexpression of cathepsin-K leads to the detachment of the cargo with a simultaneous exposition of the RGD pattern inducing the internalization of the transported drugs into the tumoral cells.

### Double targeting cellular–intracellular trafficking

Besides tissue accumulation, the preferential internalization in tumoral cells is the main goal to improve the efficacy of a drug. Endosomal scape and nanocarrier vectorization to one specific organelle may be essential for improving the therapeutic effect at low concentrations, as has been described above. The combination of subcellular targeting agents that allow for endosomal scape and regulate the intracellular trafficking and a targeting agent directed to cell membrane receptors is a promising strategy for increasing the efficacy of treatments. The presence of both an intracellular and extracellular targeting agent would drive the drug nanotransporter directly to the desired organelle. Many systems use the combination of two different targeting agents to carry out the multi-vectorization process. In other cases, the intracellular and extracellular targeting effects come from the structural properties of the nanocarriers [81]. Usually, the aim of the intracellular vectorization is oriented to nucleus or mitochondria to improve the efficacy of the transported drug for cancer treatments. Fortunately, there are intracellular vectorization motives for almost all subcellular localizations [42,82].

There are many cytotoxic drugs, such as doxorubicin, that induce cell apoptosis through intercalation with nuclear DNA. Further, gene silencing therapies based on an effective delivery of short hairpin RNA (shRNA) bearing genes for small interfering RNA (siRNA) need nuclear vectorization for enhancing the cell growth inhibition. In these cases, the goal is to drive the payload to the nucleus after selective internalization in the cytoplasm. Nuclear delivery with the HIV trans-activator of transcription (TAT) peptoid in combination with the vasculature and tumor cell membrane targeting RGD tripeptide is a novel strategy recently described. Both vectorization agents were grafted on mesoporous silicananoparticles in a random manner [72]. RGD act as tissular and cellular targeting ligand, while TAT act as CPP mediating the endosomal escape and driving the payload to the nucleus. This example reflects how the combination of two targeting agents works on three levels of vectorization, namely tissular, cellular and subcellular targeting.

**Figure 7:**
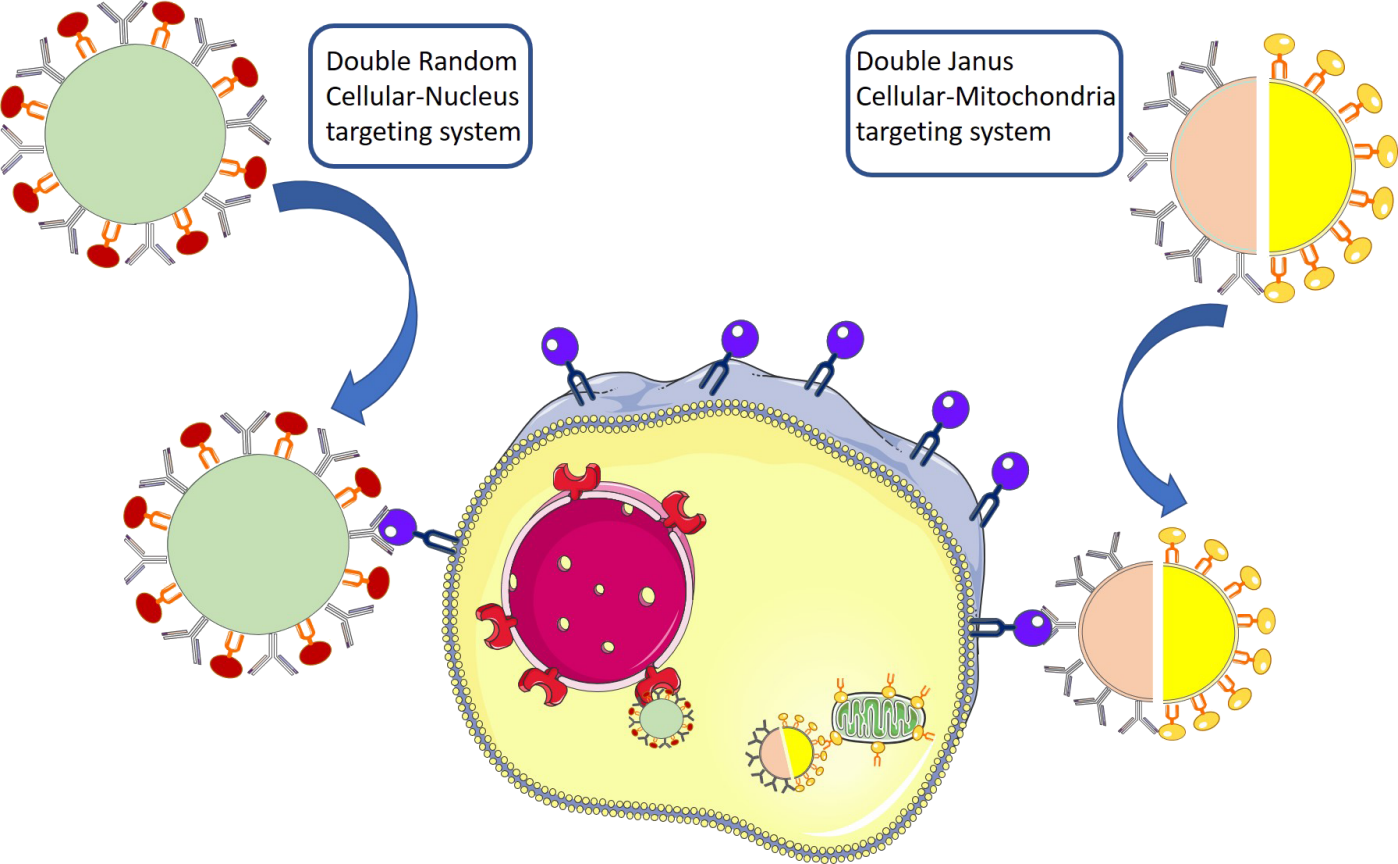
Double cell–organelle targeting strategies.

As mentioned above, there are unwanted interaction effects between two agents, especially in the case of random decoration. Janus systems have become a great alternative for including double functional targeting agents to a nanocarrier. Villegas et al. [83] recently described mesoporous Janus nanoparticles for dual targeting of tumour cells and mitochondria (Figure 7). There are multiple “mitochondriotoxic” drugs that act on mitochondria inducing cell apoptosis, such as gamitrinibs and cisplatin [84]. One of the best ways to bring molecules or nanocarriers to mitochondria is by using positively charged groups. These motives are able to escape from the early endosome and reach to the mitochondria [85–86]. One of the most commonly used cationic groups as mitochondrial targeting agent is the triphenylphosphonium cation (TTP) [87]. The vectorization agents are anchored separately on each of the hemispheres of the nanocarrier through an asymmetrisation process. One hemisphere is functionalized with folic acid as cell membrane targeting ligand and the other hemisphere is functionalized with a TPP analogue, which allows for endosomal escape and drives the nanocarrier to the mitochondria surroundings. This strategy turned out to be successful for the transportation of topotecan to the mitochondrial environment resulting in a highly efficient in vitro treatment of human prostate cancer cells. The excellent performance could be explained by the multivalence effect. The fact of having the same ligand interacting with the same receptor simultaneously without any other non-specific interaction from the other ligand maximized the interactions enhancing the particle uptake.

### Double targeting with the same motive: multivalence effect

**Figure 8:**
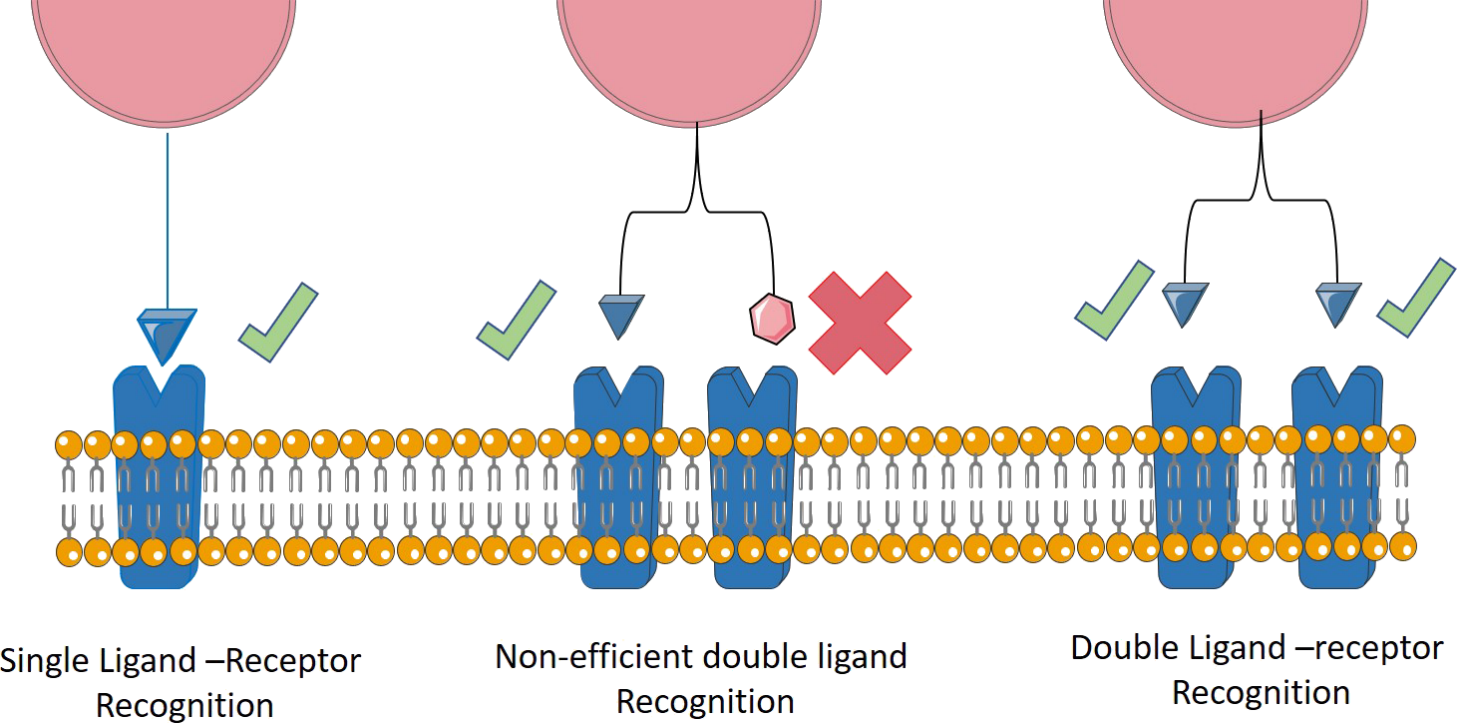
Multivalence effect in multiple targeting systems.

The design of double targeting agents with the same motive in a single system is nowadays an alternative to inefficient vectorization in the cases in which the single ligand–receptor recognition is not strong enough to induce an enhancement of the cell internalization. Jin et al. [88] developed a double linear RGD-type ligand that was evaluated in comparison to the single counterpart with regard to binding affinity and specificity to integrins in vitro and in vivo. Further, Rosca et al. [89] described effective single, double and triple systems decorated with dodecapeptide with affinity to integrins overexpressed in glioma cells. The effect of multiplying the motives in the structure sharpens the contrast of binding between cancer and healthy cells. The work demonstrated an improvement in terms of selectivity and retention in tumoral tissues overexpressing integrins. Nature uses these types of double interaction moieties as secure systems, the double interaction between ligand and receptor is present in recognition processes of, e.g., DNA and antibodies [90]. The structural flexibility and the distance between the motives yield the desired interaction with the receptor and allow for strongest interactions compared to nonspecific interactions (Figure 8).

## Conclusion

The last few decades have witnessed the emergence of nanomedicine in the oncological field. The use of nanoparticles as drug delivery systems provided unique advantages such as the possibility to transport highly lipophilic drugs and to improve the pharmacokinetic profile of these drugs, enhancing their accumulation both in the tumoral tissue and within the malignant cells. Moreover, it is even possible to control the drug release process through the incorporation of stimuli-responsive mechanisms that regulate the drug release from the nanocarrier. The incorporation of targeting moieties on the carrier surface produces a significative increase in the particle accumulation inside tumoral cells, which could improve the efficacy of the therapy due to the high amount of drug that is possible to deliver into the diseased cells, and by the reduction of side effects. Despite the encouraging results there is much work left to be done until these targeted nanocarriers fulfill the high expectations. Many of these systems have been tested employing in vitro assays, or xenograft in vivo models in the best cases. The fact that excellent results are observed with these assays does not guarantee the same behaviour in clinical trials due to the huge complexity of real tumours. It is compulsory to evaluate the real efficacy of the targeted nanodevices in more realistic scenarios such as the utilization of orthotopic animal models in which the tumour growths in its natural environment. Additionally, the efficacy of these systems should be studied employing immunocompetent animal models to study how a fully operative immune system reacts to the administered nanocarriers. In any case, we are at the beginning of nanomedicine. The excellent results obtained until now paved the way for the development of novel and more functional targeted nanocarriers that would eradicate devastating diseases in the coming future.
